# Supplementary material for: Chromosome silencing in vitro reveals trisomy 21 causes cell-autonomous deficits in angiogenesis and early dysregulation in Notch signaling
Source: Cell Rep. Author manuscript; Available in PMC 2022 Sep 23. (PMC9505374; doi:10.1016/j.celrep.2022.111174)
Supplement: 1 [file NIHMS1829260-supplement-1.pdf]

Cell Reports, Volume *40*

Supplemental information

Chromosome silencing *in vitro* reveals trisomy 21 causes cell-autonomous deficits in angiogenesis and early dysregulation in Notch signaling

Jennifer E. Moon and Jeanne B. Lawrence

SUPPLEMENTARY INFORMATION

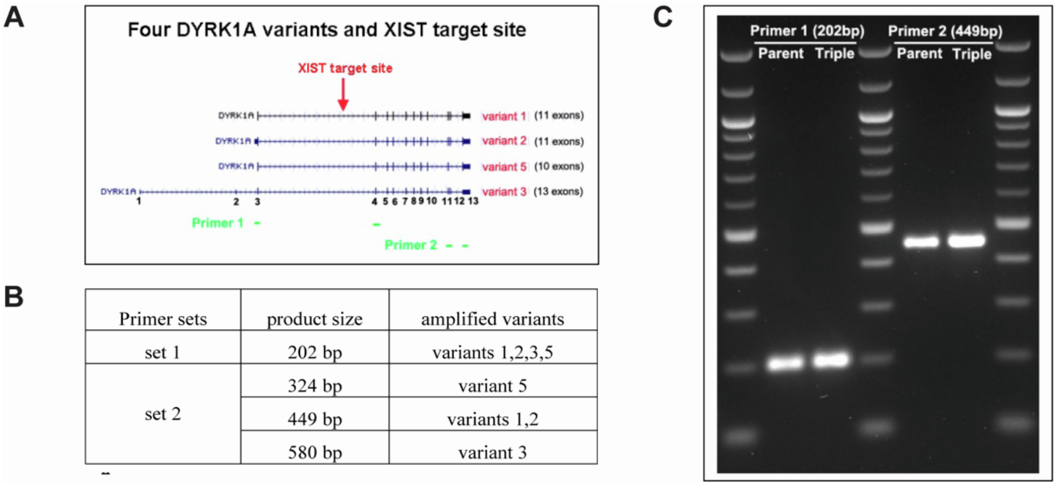

Figure S1: *DYRK1A* expression is not disrupted by XIST insertion, related to Figure 1
(A) *DYRK1A* splicing isoforms with differing 5'UTR and 3' coding regions. The *XIST* transgene (red) was inserted into the intron of variants 1, 2, 5, or intron 3 of variant 3 shown here. Primers were designed to detect for *DYRK1A* expression (green).
(B) Anticipated product sizes for each variant.
(C) RT-PCR results shows the first set of primers generates a 202 bp band, and the second set of primers generates only one 449 bp of single band in both parental and triple target lines (*XIST* inserted in all three alleles of *DYRK1A*). Sequencing results confirm the 202 bp product from the first set of primers in both lines is the sequence spanning exon 1 and exon 2 of variants 1, 2, 5, or spanning exon 3 and exon 4 of variant 3.

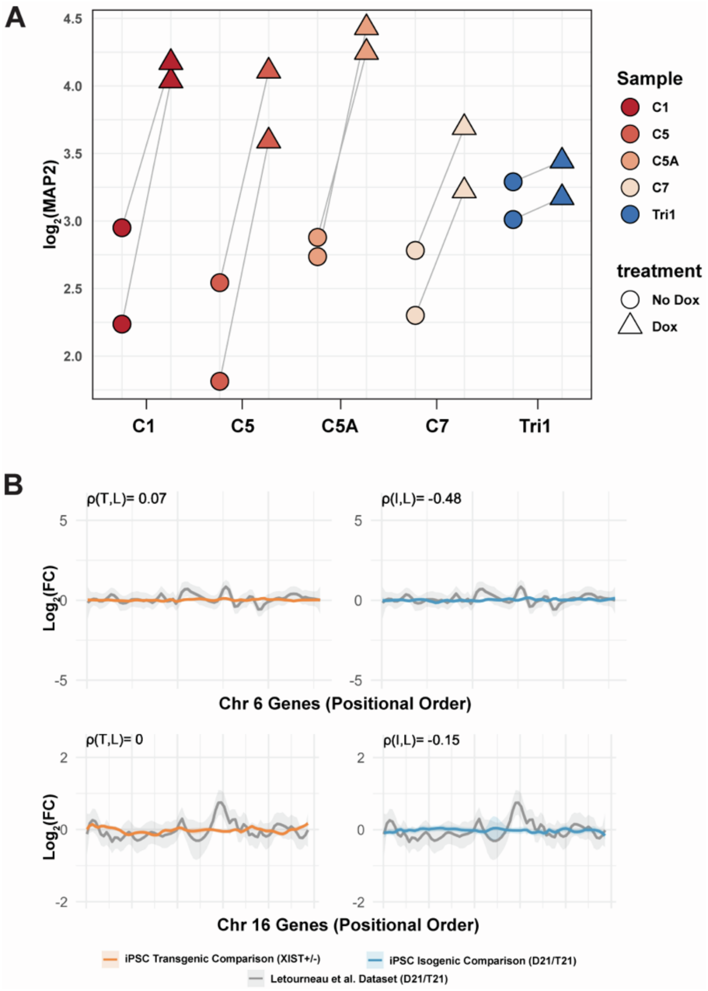

Figure S2: Gene expression patterns seen in DS iPSCs, related to Figure Figure 2
(A) MAP2 expression of all cell lines in counts per million (CPM). Non-dox treated cells are in dark gray and dox treated cells are in light gray.
(B) Representative local regression plots of chr 6 and chr 16. Letourneau et al. dataset (gray) is plotted against our transgenic or isogenic comparison. Pearson correlation (Rho) is reported above each comparison. P > 0.05.

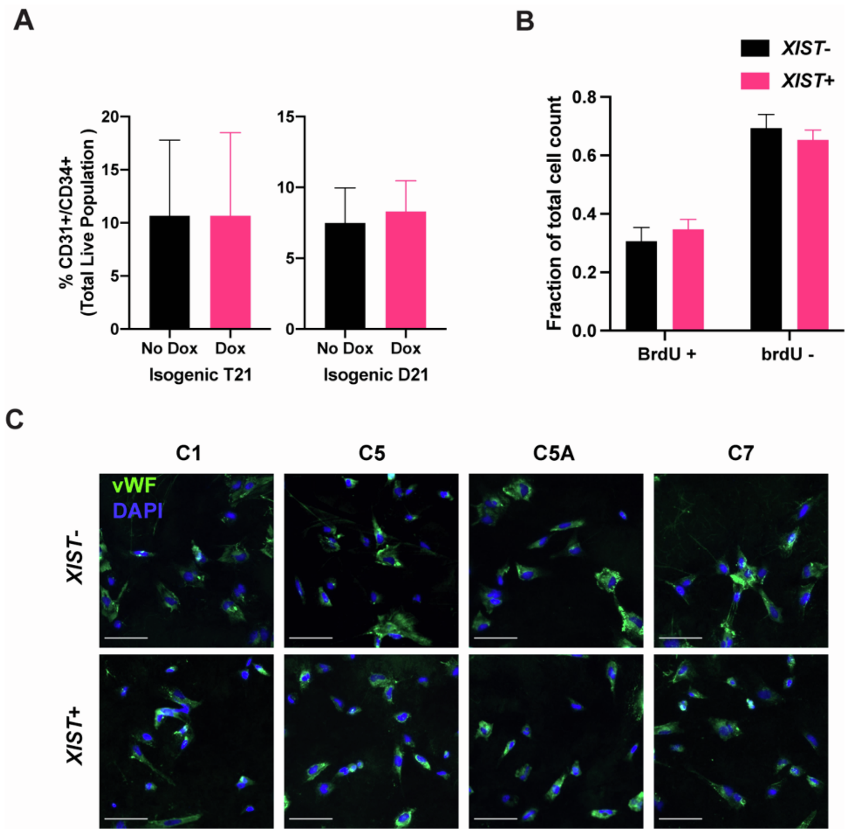

Figure S3: Characterization of XIST- and XIST+ endothelial cells, related to Figure 4

(A) Quantification of CD31+CD34+ cells in one isogenic trisomic (T21) and disomic lines (D21) with *TET3G* but lacking the *XIST* transgene. Experiment was conducted in parallel with the transgenic lines and repeated three times (mean ± SD).

(B) Representative immunofluorescence images of each condition for vWF (endothelial cell marker). Scale bar = 100 um.

(C) After endothelial progenitor cell enrichment and expansion (day 10), cells were incubated with BrdU for 2 hours. Quantification of BrdU positive and negative cells (400-500 observations per condition; n = 4, mean ± SD; paired t- test p-value = 0.02).

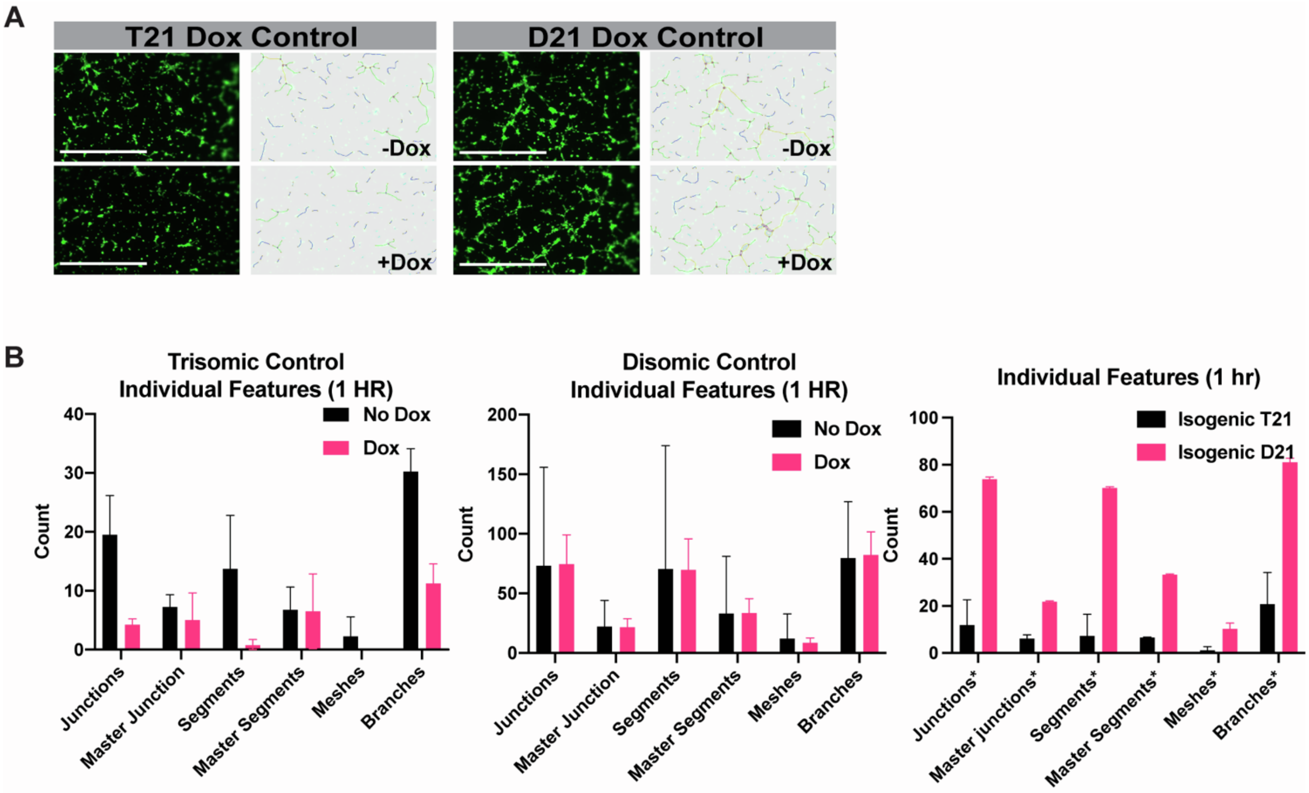

Figure S4: Isogenic Trisomy/Disomy comparison after 1 hours, related to Figure 5

(A) Representative images of tube formation after one hour of incubation in the dox control lines (Tri1 and Dis1) run in parallel with the transgenic lines seen in Figure II-5B and repeated three times. Scale bar = 1000 um.

(B) The two panels on the right are quantification of tube formation from images represented in (A) using the dox control lines (mean ± SD). The third (left) panel is the comparison between individual features detected by Angiogenesis Analyzer in one isogenic T21 (Tri1) and D21 (Dis1) sample after one hour of incubation (mean ± SD).

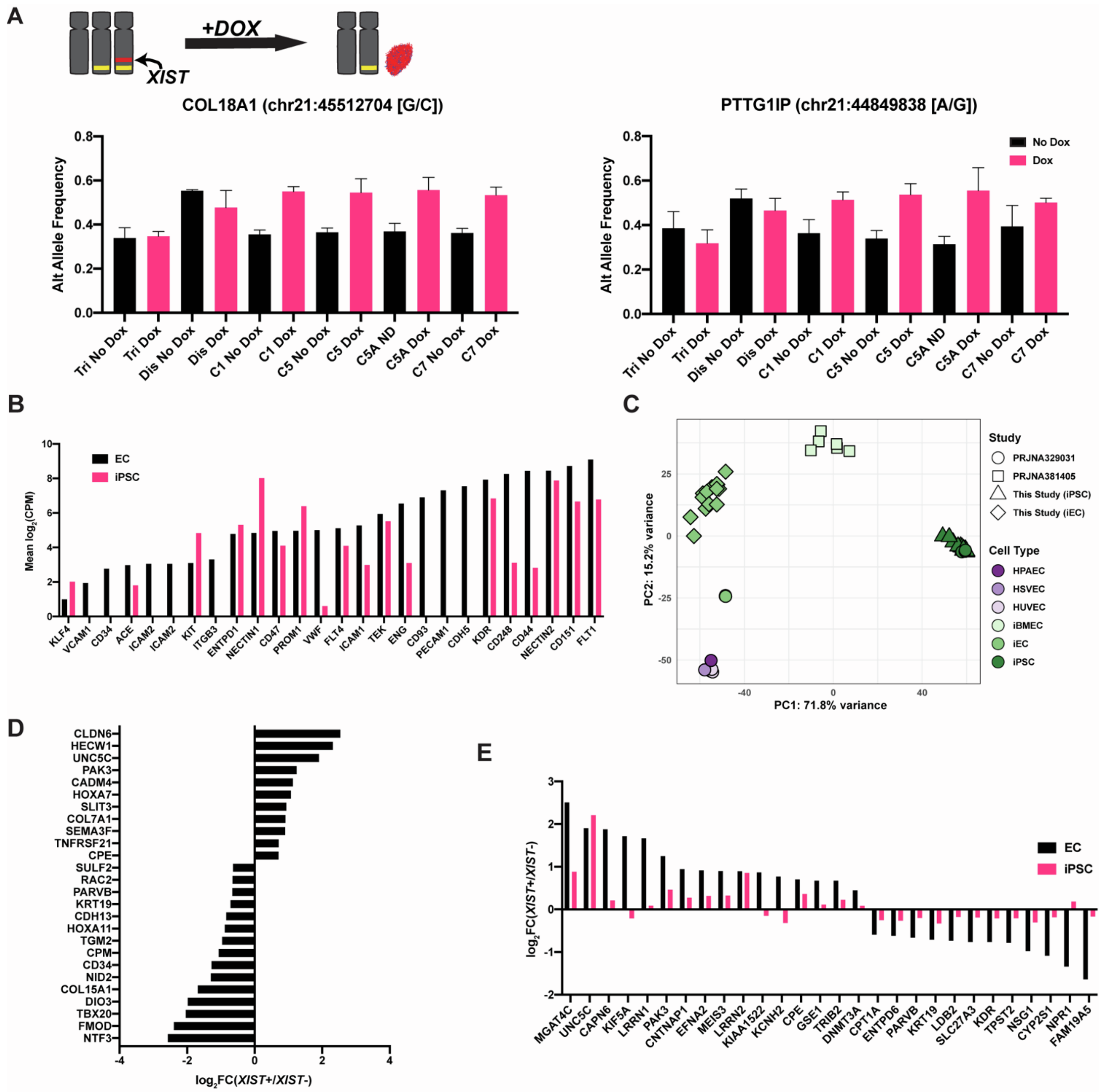

Figure S5: Gene expression analysis of iPSCs and ECs., related to Figure 6

(A) Illustration of allele frequency changes due to *XIST*-mediated silencing (the allele on the extra chr21 is depicted in yellow and *XIST* locus in red) and two representative SNPs from the endothelial data set after treatment of dox. Each graph shows the gene symbol, its chromosomal position, and reference/alternate alleles. Both genes are ~8 Mb away from the *DYRK1A* locus.

(B) Mean log2 (CPM) expression of common endothelial cell markers are plotted for both EC (black) and iPSC (pink) datasets.

(C) Principal component analysis of the endothelial cells generated in this study against publicly available RNA-seq datasets from iPSC derived endothelial cells (iEC and iBMEC) and primary endothelial cells (HPAEC, HSVEC, HUVEC). iECs generated from this study clustered well with other ECs across principal component 1.

(D) The logFC of DEGs relating to cell migration, adhesion, and extracellular matrix (FDR < 0.05).

(E) Genes differentially expressed in both iPSC and EC datasets after silencing the extra chr21 (FDR < 0.05).

Table S1: Differentially expressed chr21 genes in each RNA-seq dataset after chromosome 21 silencing (FDR < 0.05), related to Figure 3 and 6

iPSC Dataset	ABCG1, ADARB1, AP000688.2, AP001505.1, APP, ATP5PF, ATP5PO, B3GALT5, B3GALT5-AS1, BACE2, BACH1, BRWD1, C21orf91, C2CD2, CBR1, CBR3, CFAP298, CHAF1B, CLIC6, COL6A2, CRYZL1, CSTB, CXADR, DIP2A, DONSON, DOP1B, DYRK1A, ETS2, FAM207A, FP565260.1, GABPA, GART, GATD3A, HLCS, HMGN1, HSPA13, HUNK, IFNAR1, IFNGR2, ITSN1, JAM2, KCNJ6, LINC00205, LINC00649, LINC02575, LRRC3, LSS, LTN1, MCM3AP, MIS18A, MORC3, MRPL39, MRPS6, MX1, MX2, N6AMT1, NDUFV3, NRIP1, PAXBP1, PCNT, PDE9A, PDXK, PFKL, PKNOX1, POFUT2, PRDM15, PRMT2, PSMG1, PTTG1IP, PWP2, RCAN1, RIPK4, RRP1, RRP1B, SCAF4, SETD4, SLC19A1, SLC37A1, SLC5A3, SOD1, SON, SPATC1L, SUMO3, SYNJ1, TIAM1, TMEM50B, TMPRSS2, TRAPPC10, TSPEAR, TTC3, UBE2G2, URB1, USP16, USP25, VPS26C, WDR4, ZBTB21
EC Dataset	AGPAT3, APP, ATP5PO, B3GALT5, B3GALT5-AS1, BACE2, BRWD1, BTG3, C2CD2, CBR1, CBR3, CCT8, CFAP298, COL18A1, COL6A1, COL6A2, CRYZL1, CSTB, CYYR1, ETS2, EVA1C, FAM207A, HLCS, HMGN1, IFNAR1, IFNGR2, ITSN1, LINC00205, MIS18A, MORC3, MRPS6, N6AMT1, NCAM2, NDUFV3, NRIP1, PAXBP1, PDXK, PFKL, PIGP, PSMG1, PTTG1IP, RRP1, RRP1B, RWDD2B, SAMSN1, SCAF4, SOD1, SUMO3, SYNJ1, TIAM1, TTC3, UBE2G2, URB1, USP16, USP25, VPS26C, ZBTB21
